# Light‐Emitting Microinlaid Spots Produced through Lateral Phase Separation by Means of Simple Single‐Inkjet Printing

**DOI:** 10.1002/smsc.202200017

**Published:** 2022-05-09

**Authors:** Byoungchoo Park, Jaewoo Park, Wonsun Kim, Seo Young Na, Yoon Ho Huh, Mina Kim, Eun Ha Choi

**Affiliations:** ^1^ Department of Electrical and Biological Physics Kwangwoon University Wolgye-Dong Seoul 01897 South Korea; ^2^ Department of Plasma-Bio Display Kwangwoon University Wolgye-Dong Seoul 01897 South Korea

**Keywords:** convective and marangoni flows, inkjet printing, light-emitting devices, microinlaid spots, micropatterning, microfluid flow-induced phase separation, poly(4-vinylpyridine)

## Abstract

High‐performance solution‐processable light‐emitting diodes (LEDs) attract much research interest due to the very high complexity of conventional vacuum‐processed LEDs. A simple single‐inkjet‐printing process using a phase‐separable material combination is presented. With single‐inkjet printing of an ink containing semiconducting compounds on a phase‐separable insulating layer, convective and Marangoni flows in sessile droplets can produce microinlaid spots through the site‐selective etching of the insulating layer and the simultaneous self‐filling of the semiconductors in the etched vacancies. As a proof of concept, microinlaid organic LEDs (OLEDs) with a spatial resolution of ≈200 dpi in a phase‐separable poly(4‐vinylpyridine) layer without any conventional preformation of bank‐like structures are produced. The fabricated green microinlaid OLEDs exhibit excellent performance with maximum brightness of ≈13 000 cd m^−2^ and maximum efficiency of ≈14.2 cd A^−1^. Moreover, large‐area inkjet‐printed OLEDs are simply realized using the microinlaid spot arrays. These results demonstrate that the inkjet‐inlay structure is a promising candidate for high‐performance next‐generation solution‐processable LEDs.

## Graphical abstract

A light‐emitting inkjet‐inlaid spot is produced utilizing an appropriate material combination of a phase‐separable insulating polymer and functional semiconducting compounds for the simple and reliable fabrication of high‐performance small‐molecular micro‐organic light‐emitting diode (OLED) pixel arrays. Due to the self‐creation of high‐quality microinlaid spots, bright, efficient, and large‐area micro‐OLED pixel arrays are easily realized without any misalignment by the single‐inkjet‐printing process.

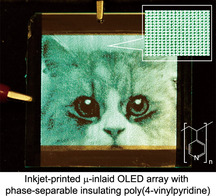

## 1 Introduction

Inkjet printing has been the subject of considerable attention over the past few decades, as its great potential offers high‐quality patterning technologies by printing very small droplets, allowing high resolutions, rapid processing, and low material waste.^[^ 1, 2, 3, 4, 5, 6, 7, 8, 9 ^]^ Due to such advantages, the effective micropatterning of functional components has been demonstrated for various applications in the display,^[^ 10, 11, 12 ^]^ semiconductor,^[^ 13, 14, 15, 16, 17, 18, 19 ^]^ and biomedical industries,^[^ 20, 21 ^]^ as well as in the conventional graphics industry. In relation to these applications, a number of studies of inkjet printing have sought to realize uniform pixel formation and patterning for solution‐processable light‐emitting diodes (LEDs) that utilize not only organic semiconducting materials^[^ 10, 11, 12, 22, 23, 24, 25, 26, 27, 28, 29, 30, 31, 32, 33 ^]^ but also quantum dots (QDs).^[^ 34, 35, 36, 37 ^]^ As recent examples, by the precise deposition of droplets using the drop‐on‐demand inkjet‐printing technique, thin red‐ (R), green‐ (G), blue‐ (B), and/or white‐emitting material layer (EML) pixels were fabricated for high‐performance light‐emitting pixel arrays of organic LEDs (OLEDs) as well as QD LEDs (QD‐LEDs).^[^ 29, 30, 31, 32, 33, 34 ^]^ Nevertheless, in many of these examples, prior to the inkjet‐printing deposition step, the substrates were usually prepatterned with insulating polymeric walls of acrylate or polyimide materials surrounding each pixel site in the form of a bank‐like structure, into which the ink droplets can be held,^[^ 29, 30, 31, 32, 33, 34 ^]^ except for several examples without such prepatterned structures.^[^ 12, 22, 23, 24 ^]^ Such a prepatterning process, normally including complex photolithographic procedures, has the potential to contaminate the surface of the substrate, unavoidably deteriorating device performance capabilities.

Meanwhile, inkjet printing has also been developed as a subtractive tool capable of site selectively removing small areas of predeposited layers, that is, inkjet etching.^[^ 38, 39, 40, 41, 42, 43 ^]^ In the inkjet‐etching process, a solvent in the droplet locally dissolves a predeposited polymer layer, resulting in a crater‐shaped via‐hole that forms due to the redeposition of the dissolved polymer on the contact line at the edge of the droplet. The mechanism of this inkjet‐etching process for via‐hole formation is commonly known as the coffee‐ring effect due to the microfluid flows in a sessile droplet.^[^ 38, 39, 40, 41, 42, 43 ^]^ Such an inkjet‐based selective etching process has several advantages over conventional complex photolithographic etching processes and can therefore allow a simple maskless, photoresist‐free, and cost‐saving patterning process feasible for creating open via‐holes in electronic devices, biochips, and micropatterned cell arrays.^[^ 38, 39, 40, 41, 42, 43 ^]^ In particular, based on these advantages, inkjet‐etched open via‐holes were also used as bank‐like walls not only for thin‐film transistors^[^ 38, 41 ^]^ but also for inkjet‐printed micro‐OLEDs (μ‐OLEDs),^[^ 42, 43 ^]^ which were produced by the subsequent inkjet printing of light‐emitting materials into the inkjet‐etched open via‐holes. During these subsequent inkjet‐printing processes, however, several critical pixel defects can arise due to misalignments between the inkjet‐etched open via‐holes and the subsequent inkjet‐printed material arrays. Such pixel defects due to misalignments can thwart deliberate device fabrication efforts.

To overcome the aforementioned issues that affect these subsequent inkjet‐printing processes, a new type of direct inkjet‐printing technique is required, in which the two separate etching and filling steps should be combined into a single process, that is, single‐inkjet printing, to serve as a site‐selective spatial etching and simultaneous filling tool. To achieve this goal, a functional layer that plays multiple roles can be introduced in the inkjet‐printing process. As a preliminary example, a simple maskless inkjet‐patterned OLED that included a thin insulating layer of poly(methyl methacrylate) (PMMA) was suggested.^[^ ^44^ ^]^ Despite its simple fabrication process, it was ineffective; due to the PMMA polymer remaining inside the inkjet‐printed spots of the light‐emitting functional materials, the inkjet‐printed OLED based on PMMA exhibited relatively poor device performance. The PMMA‐based inkjet technology thus remains associated with difficulties in regard to improving the low quality and/or low purity levels of functional materials in printed spots.

In this study, simple and practical single‐inkjet printing of a simultaneous etching and filling process is reported for fabricating high‐quality inkjet‐printed light‐emitting pixel array patterns. In this process, we utilize an appropriate material combination of phase‐separable functional semiconducting and insulating compounds having different solubility properties. An ink solution including the functional semiconducting compound of small‐molecular 4,4′‐bis(*N*‐carbazolyl)‐1,1′‐biphenyl (CBP) is inkjet printed onto the phase‐separable insulating compound layer of poly(4‐vinylpyridine) (P4VP). Measurements and an analysis are conducted to characterize the structure of the printed spots, with these results showing a high‐quality microinlaid spot structure of CBP in the insulating P4VP layer. The basic mechanism behind the formation of the microinlaid spot structure is described in terms of the site‐selective etching of the P4VP layers together with the simultaneous filling of semiconducting CBP molecules into the etched vacancies based on lateral phase‐separation behavior.^[^ ^45^ ^]^ In the following section, the single‐inkjet‐printing process is applied to produce light‐emitting microinlaid spot pixel arrays. Inkjet‐inlaid μ‐OLED spot arrays with a pixel resolution of about 200 dpi are easily fabricated with using light‐emitting compounds, offering more freedom for fabricating light‐emitting displays with regard to different device architectures, deposition methods, and panel structures. Further perspectives are also discussed with respect to the production of single‐inkjet‐printed light‐emitting devices of μ‐OLEDs as well as μ‐QD‐LEDs (or nano‐LEDs).^[^ 34, 37, 46, 47 ^]^ To the best of our knowledge, this is the first time the simplest single‐inkjet process is reported for high‐performance microinlaid OLED pixel arrays using a phase‐separable material combination.

## 2 Results and Discussion

### 2.1 Microinlaid Spot Structure: Single‐Inkjet‐Printed Spots of Semiconducting CBP in Phase‐Separable Insulating P4VP Layers

**Figure 1:**
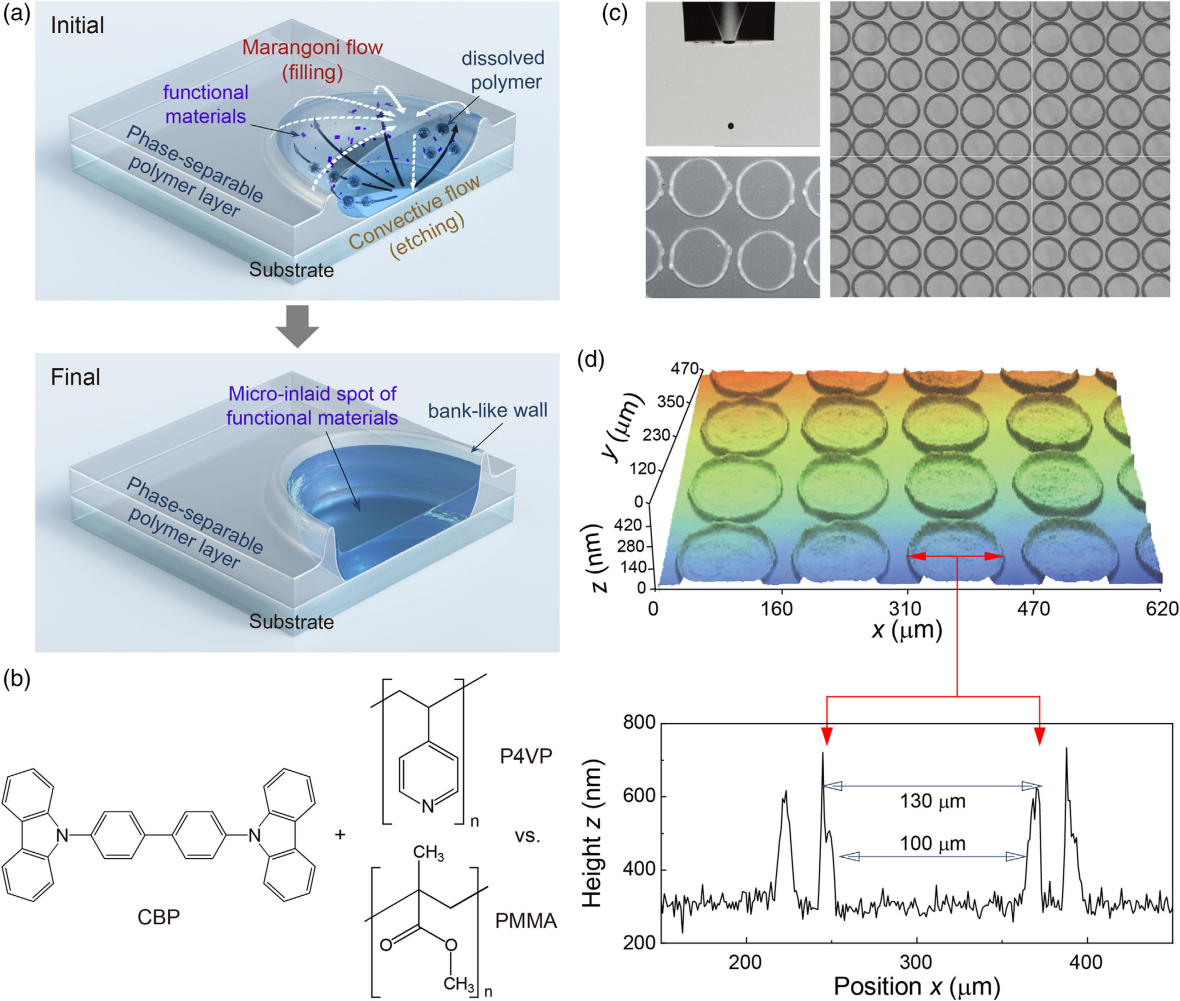
a) Schematic illustrations of the single‐inkjet‐printing technique studied here: a sessile droplet of a functional ink is inkjet printed on a phase‐separable insulating polymer layer (upper). The solvent in the droplet locally dissolves the polymer layer, and the dissolved polymers are transported to the edge of the droplet by the convective flows, thus removing the insulating polymer from the center region and causing it to settle around the edges of the droplet. Meanwhile, simultaneously, as the solvent evaporates, the functional solutes conveyed by the convective flow return to the center region by a Marangoni flow and begin to precipitate and become deposited on the entire printed spot area, eventually resulting in the formation of a microinlay structure containing high‐quality functional solute materials in the printed spot region in the insulating layer through the flow‐induced lateral phase separation (lower). b) Molecular structures of the functional materials used in this study. c) A photographic image (left upper) of a flying droplet ejected from an inkjet nozzle with an orifice diameter of 50 μm using an ink containing small‐molecular CBP in chloroform. SEM (left lower) and optical microscopy (right) images of a rectangular array of inkjet‐printed spots on stacked layers of PVK/P4VP by the single‐inkjet‐printing process at 180 dots per inch (dpi). d) 3D surface image (upper) and corresponding 2D profile (lower) of a single‐inkjet‐printed spot array. Self‐organized circular bank‐like walls around the inkjet‐printed spots are clearly shown in the 3D surface image. Each droplet forms a single circular microspot pixel, of which the inner diameter is ≈100 μm.

The single‐inkjet‐printing technique investigated in this work is based on the use of a material combination of laterally phase‐separable functional compounds. The phase‐separable material combination used herein includes a small‐molecular organic semiconducting compound as a functional solute in an ink solution together with a phase‐separable polymer for an insulating layer on a target substrate to form the inkjet‐inlaid spot structure, as shown in **Figure** **1**a. First, prior to inkjet printing, a homogeneous and thin insulating phase‐separable polymer layer is prepared on the underlying functional layers, for example, a hole‐injecting layer (HIL) and/or hole‐transporting layer (HTL), on a transparent indium‐tin‐oxide (ITO) substrate. Next, droplets of the ink containing the functional solute are inkjet printed onto the insulating polymer layer. After inkjet printing, the solvent in the sessile droplet begins to dissolve and etch the polymer layer. Then, the dissolved polymer is delivered to the edge of the droplet (the contact line of the droplet) by the convective and/or capillary flows of the solvent and consequently redeposited at the edge of the droplet,^[^ 7, 8, 38, 39, 40, 41, 42, 43, 48 ^]^ eventually constructing a hole‐like vacancy in the polymer layer (upper panel of Figure 1a). Meanwhile, simultaneously, as the solvent in the sessile droplet evaporates, only the phase‐separated functional solute molecules begin to recirculate from the edge to center of the droplet via Marangoni flows.^[^ 17, 18, 39, 48 ^]^ Subsequently, the solvent in the droplet evaporates further and the functional solute molecules are extracted and deposited on the printed area, finally filling the hole‐like vacancy. Thus, high‐quality spots of the functional solute can be (in situ) formed with the shape of a filled crater in the insulating polymer layer, that is, the inkjet‐inlay structure. Hence, a well‐defined microinlay structure of these types of functional solute materials can be self‐organized without serious comixing of the phase‐separable insulating polymer inside the inkjet‐printed spot region (lower panel of Figure 1a).

Herein, to realize the microinlaid spot array, we used CBP, a small‐molecular semiconducting host compound with a wide bandgap as the functional solute in the ink solution, together with P4VP, a relatively hydrophobic polymer as the insulating layer, which exhibits different phase behavior from the typical polymer PMMA (Figure 1b).^[^ ^49^ ^]^ Figure 1c shows a photograph of the flying droplet profile of the ink containing CBP in chloroform (diameter: ≈65 μm) when it is ejected through an inkjet nozzle with an orifice diameter of 50 μm. As shown in the figure, drop formation of the ink is highly stable without any significant clogging of the nozzle, mainly due to the low viscosity (≈0.6 cp) of the ink solution containing the small‐molecular compound. The figure also shows scanning electron microscopy (SEM) and optical microscopy images of a rectangular array of single‐inkjet‐printed spots on stacked layers consisting of an insulating P4VP layer and a hole‐transporting poly(9‐vinylcarbazole) (PVK) layer (PVK/P4VP). As shown in the figure, regularly separated and circular microspots were readily formed on the PVK/P4VP stacked layers; the inner diameter of the microspot was ≈100 μm, which is nearly twice as large as that of the flying ink droplet. We subsequently investigated the microscopic surface morphologies of the single‐inkjet‐printed spots using a 3D surface profiler (Figure 1d). In the figure, the surface profiles show clear microspots, well separated and surrounded by self‐organized bank‐like walls, exhibiting the shape of the filled crater. In the inner region of the printed spot, the measured root mean square (RMS) surface roughness was ≈2.4 nm, similar to that (≈2.3 nm) of a conventional spin‐coated CBP layer. Thus, the surface profiles investigated herein confirmed that the topography of the inner region of the printed spots is fairly smooth and uniform without any serious defects, such as pinholes and/or incomplete coverage areas.

### 2.2 Confocal Raman Spectra of a Single‐Inkjet‐Printed CBP Spot in a Phase‐Separable Insulating P4VP Layer

**Figure 2:**
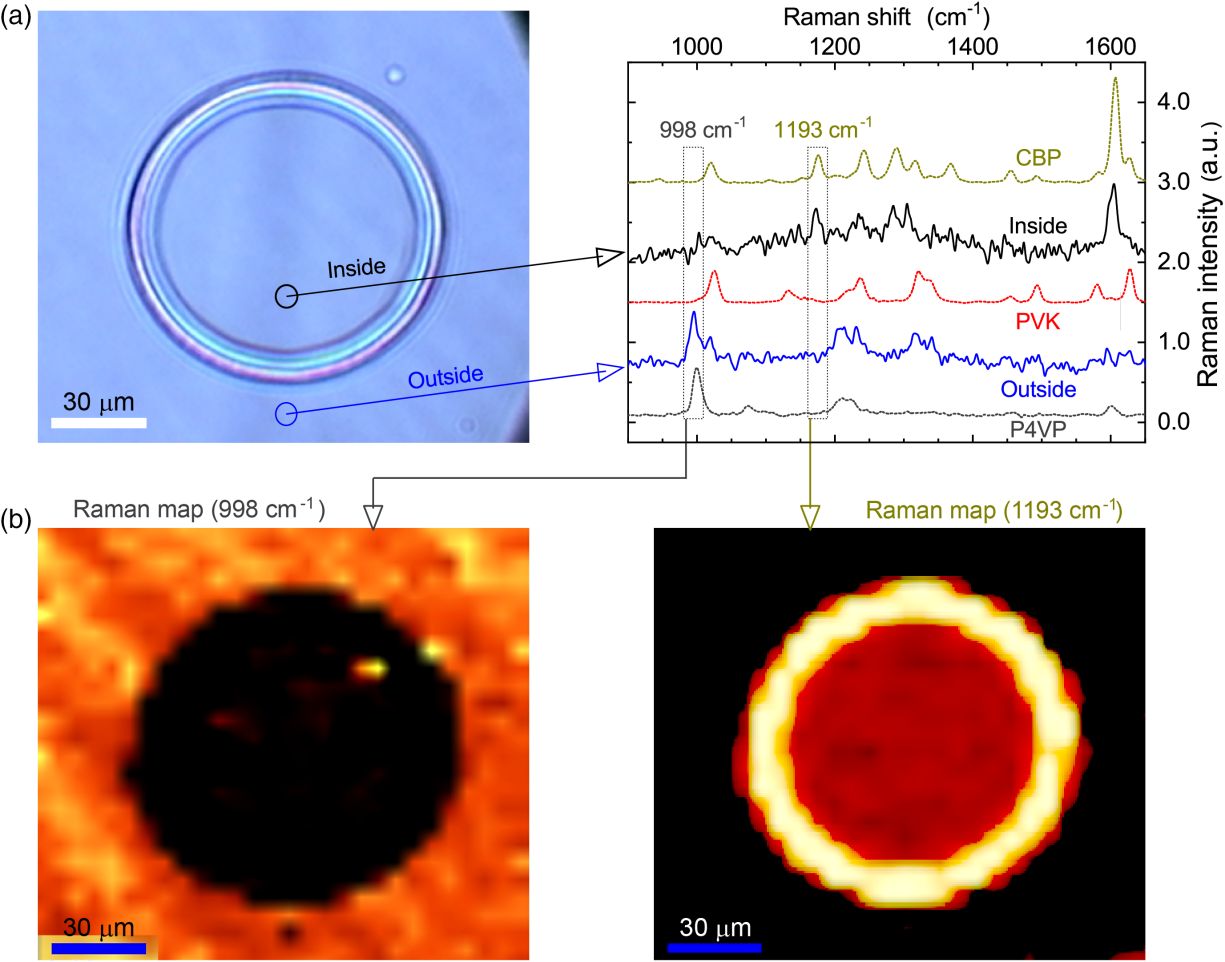
a) Left: Optical microscopy image of a single‐inkjet‐printed spot onto stacked layers consisting of an insulating P4VP layer and a PVK layer (P4VP/PVK) using an ink containing small‐molecular CBP in chloroform. Right: confocal micro‐Raman spectra from the inside and outside of the inkjet‐printed spot under 632.8 nm excitation (5 mW). For comparison, Raman spectra from pure compound layers of CBP, PVK, and P4VP are also shown in the figure. b) Raman images obtained by integrating Raman intensities for the ring‐breathing mode (998 cm^−1^) of pyridine groups in P4VP (left image) and for the in‐plane deformation mode (1193 cm^−1^) of the C—H bond in CBP (right image) for the single‐inkjet‐printed spot.

Next, to identify the composition of the single‐inkjet‐printed spots, confocal Raman spectra were measured at several different positions in the printed spot region upon optical excitation at 632.8 nm, with these results then compared (**Figure** **2**). The observed spot in each case was also produced by single‐inkjet printing using the ink containing CBP in chloroform on stacked layers of P4VP/PVK on a glass substrate. To increase the confocal Raman intensity levels, the glass substrate used was predeposited with a 50 nm‐thick film of Ag. An optical microscopy image of the single‐inkjet‐printed spot is shown in the panel on the left of Figure 2a. A clear ring‐shaped image surrounding a circular area inside the spot can be clearly observed. Here, to investigate the structural fingerprints, the confocal Raman spectra were measured from the inside as well as the outside regions of the printed spot, as presented in the panel on the right in Figure 2a. As also shown in the Raman spectra from the pure compound layers of CBP, PVK, and P4VP, the ring‐breathing Raman mode of pyridine groups at 998 cm^−1^ is observed only in the spectra of P4VP,^[^ ^50^ ^]^ while the in‐plane deformation mode of the C—H bond at 1193 cm^−1^ is observed only in the spectra of CBP.^[^ ^51^ ^]^ Thus, these two Raman modes at 998 and 1193 cm^−1^ are precise indications that can be used to determine the existence of both P4VP and CBP, respectively, in the inkjet‐printed spots. Using these two unique characteristic Raman modes, the confocal Raman spectra from the inside and outside regions of the single‐inkjet‐printed spot were compared with each other: a P4VP peak at 998 cm^−1^ is clearly found outside the printed spot, whereas it is negligible inside the printed spot, while the CBP peak at 1193 cm^−1^ is found only inside the printed spot.

The measured confocal Raman observations are also visualized in the Raman images, as shown in Figure 2b. The Raman image of the integrated intensity for the 998 cm^−1^ mode of P4VP (left panel in Figure 2b) evidently shows a circular dark‐colored area, the shape and diameter of which are identical to those of the inkjet‐printed spot shown in the optical image (Figure 2a). Thus, the circular dark area in Raman mapping indicates the clear etching of the P4VP layer, providing evidence that there remains a nearly negligible amount of the P4VP polymer inside the entire spot area. On the other hand, outside the printed spot, a fairly homogeneous layer of the P4VP polymer is left unchanged. This result proves that an apparent hole‐like spatial vacancy is formed inside the printed spot region through the P4VP layer. In contrast, the Raman image of the integrated intensity for the 1193 cm^−1^ mode of CBP (the right panel in Figure 2b) reveals that the functional CBP material settled only inside the spot area overall, that is, the complete filling of the hole‐like vacancy in the P4VP layer. Thus, from these Raman results, the insulating P4VP is completely etched away, while the semiconducting CBP fully fills the etched vacancy, forming the microinlay spot structure of CBP in the P4VP layer.

It is noteworthy that in the Raman image showing the 998 cm^−1^ mode, the small and bright top‐right point inside the circular dark area is due to noise spikes in the Raman spectra (Figure S1, Supporting Information). Note also that the bright intensity at the edge of the printed spot in the Raman image of the 1193 cm^−1^ mode indicates accumulated CBP molecules at the edge, mainly caused by the weak Marangoni flows. (Marangoni flows controlled by the blended solvents in the inks can reduce the inhomogeneous accumulation of CBP at the edge of spot. However, this is beyond the scope of this report, and further details about controlled and tunable Marangoni flows in this context will be reported elsewhere.) Moreover, it is important to note that the structural characteristics of the microinlay spot in the phase‐separable P4VP layer are quite different from those of previous inkjet‐printed spots on PMMA layers.^[^ ^44^ ^]^ For the PMMA insulating layer, a confocal Raman study shows that a large amount of the PMMA polymer still remains inside the inkjet‐printed spot, forming a blended mixture with CBP (Figure S2, Supporting Information), in contrast to the microinlay structure studied here, as mentioned earlier.

For a deeper understanding of the mechanism of microinlay spot formation, single‐inkjet printing is considered as a microfluid flow‐induced phase‐separation process of the functional materials used here. To assess the phase‐separation behaviors of these materials, the Flory–Huggins interaction parameters, *χ*s, are analyzed, as *χ* is an important parameter when evaluating the phase‐separation strength or immiscibility between two components in a blended system.^[^ 52, 53, 54 ^]^ In principle, a more positive *χ* value indicates that the mixing process of two components is energetically unfavorable from an enthalpic viewpoint, making their phase separation easier. The *χ* value for blended materials *i* and *j* can be evaluated using the following relationship^[^ 52, 53, 54 ^]^

(1) $$\chi_{i-j}=\frac{\sqrt{V_{i}V_{j}}}{RT}{\left(\delta_{i}-\delta_{j}\right)}^{2}$$

where *V*_*i*_, *δ*_*i*_, *R*, and *T* are the molar volume, the solubility parameter of component *i*, the gas constant, and the temperature, respectively. Herein, the molar volumes of CBP, P4VP, and PMMA compounds are 324.8, 107.7, and 83.7 cm^3^ mol^−1^, respectively. The solubility parameters, *δ*_s_, of the compounds are listed in **Table** **1**, which were retrieved from literature.^[^ 55, 56, 57, 58 ^]^ With these *δ* values, the *χ* values can be evaluated using Equation (1) for the combinations of the functional compounds used.

**Table 1:** Comparison of the Hansen solubility parameters (*δ*) of the functional compounds used in this study (at *T* = 300 K)

Compounda)	*δ*_D_ [MPa^0.5^]	*δ*_P_ [MPa^0.5^]	*δ*_H_ [MPa^0.5^]	*δ* [MPa^0.5^]	Ref
CBP	21.5	4.9	5.4	22.7	[55]
P4VP	18.1	7.2	6.8	19.0	[56]
PMMA	18.6	10.5	7.5	22.7	[57]
PVK	–	–	–	20.1	[58]

a) *δ*_D_: dispersion solubility parameter; *δ*_P_: polar solubility parameter; *δ*_H_: hydrogen bonding solubility parameter. *δ* = (*δ*_D_^2^ + *δ*_P_^2^ *+ δ*_H_^2^)^0.5^.

The evaluation of the *χ* values shows that the material combination of CBP and P4VP provides a large positive *χ* value, with *χ*_CBP‐P4VP_ being ≈0.33, which can induce great segregation between CBP and P4VP. In contrast, the combination of CBP and PMMA results in a small *χ* value of *χ*_CBP‐PMMA_ equal to nearly 0.00, which is likely to be less phase separable and comixable, indicating that PMMA can be a good polymer binder for CBP. From this Flory–Huggins analysis, the material combination of CBP and P4VP used in this study can induce phase separation much easily than the typical combination of CBP and PMMA. Thus, such easy phase‐separation behavior between CBP and P4VP can effectively induce the self‐organization of the high‐quality microinlaid spot structure of CBP in the P4VP layer. In addition, it should be noted that the PVK layer in the underlying stacked layers of PVK/P4VP may also support the formation of the microinlaid spot structure of printed CBP. The solubility parameter of PVK is ≈20.1 MPa^0.5^, between those of P4VP and CBP.^[^ ^58^ ^]^ Accordingly, on the PVK layer, P4VP and CBP are readily phase separable not only laterally from each other but also vertically from the underlying PVK layer; thus, the PVK layer can also support the formation of the microinlay structure of inkjet‐printed CBP spots in the P4VP layer.

### 2.3 Microinlaid QD Spots in a Phase‐Separable P4VP Layer

Next, in addition to the small‐molecular organic semiconducting compound of CBP, other types of functional materials, in this case fluorescent semiconducting QDs, were also examined for the formation of microinlaid spot arrays in the phase‐separable insulating layer. For the semiconducting QDs, hydrophobic green‐emitting Zn–Cu–In–S/ZnS (ZCIS/ZnS) core/shell QDs were investigated.^[^ ^59^ ^]^ A well‐dispersed suspension of ZCIS/ZnS QDs in chloroform was single inkjet printed on prepared substrates at 200 dpi using an inkjet nozzle with an orifice diameter of 50 μm, after which the printed QD spot patterns were investigated using the optical microscope and the 3D surface profiler.

**Figure 3:**
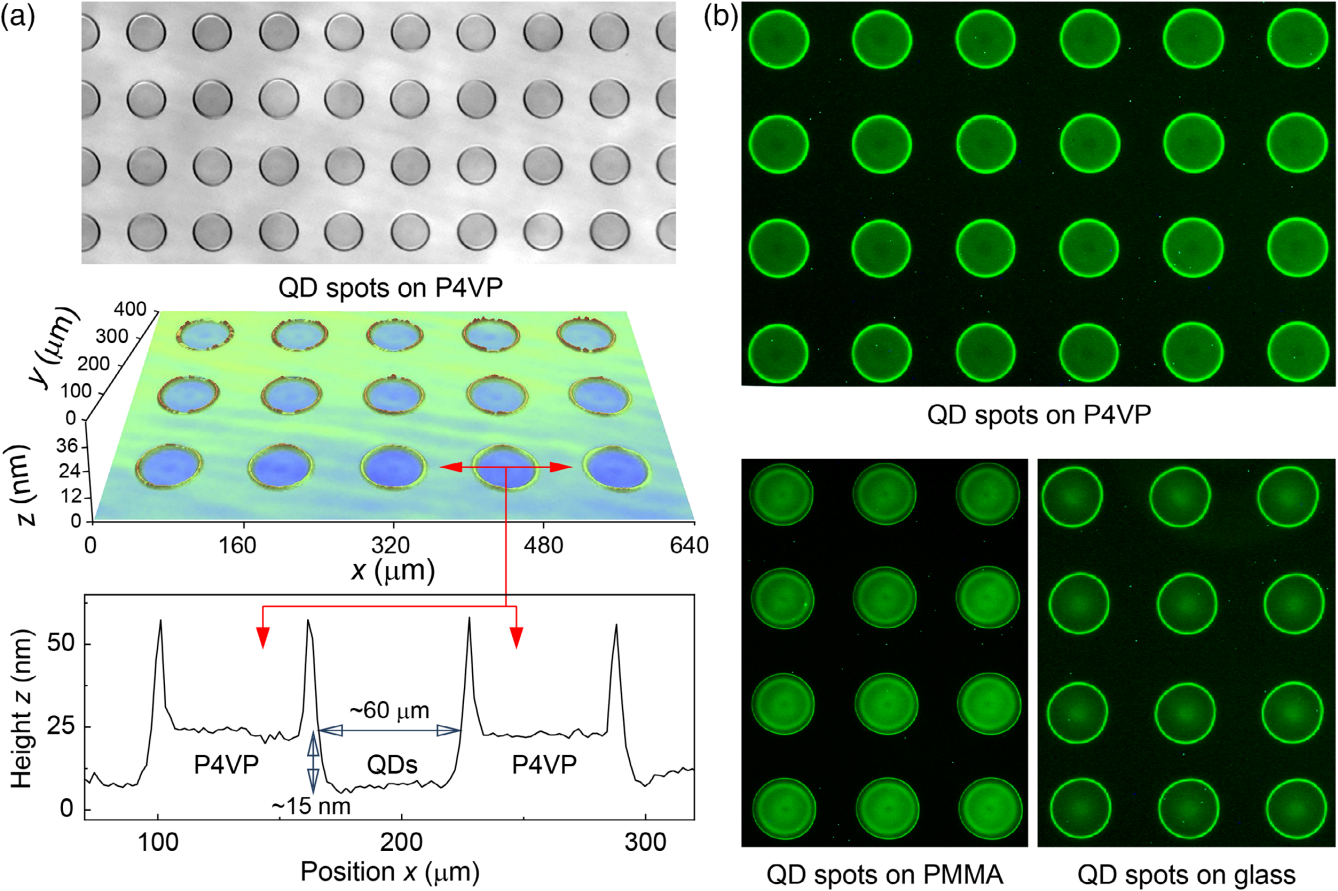
a) Optical microscopy image (upper), 3D surface image (middle), and 2D profile (lower) of a rectangular spot array of single‐inkjet‐printed Zn–Cu–In–S/ZnS (ZCIS/ZnS) core/shell QDs at 200 dpi on stacked layers of PVK/P4VP (50/30 nm) with an orifice diameter of 50 μm. Self‐organized circular inkjet‐inlaid spots are shown in the surface profile images. Each droplet forms a circular microspot pixel with a diameter of ≈60 μm. b) Fluorescence microscopy images of rectangular spot arrays of inkjet‐printed ZCIS/ZnS QDs on stacked layers of PVK/P4VP (upper), on a comparative 30 nm‐thick PMMA layer (lower left), and on a bare glass substrate (lower right).

**Figure** **3**a shows the observed height profiles of the printed QD spots on a 30 nm‐thick P4VP layer. In the figure, the height profiles for the printed spots exhibit the shape of a filled microcrater structure with a low‐lying inner region (diameter: ≈60 nm, depth: ≈15 nm) with a flat and smooth surface (RMS surface roughness: ≈1.1 nm). Notably, the printed spots were well separated and patterned with self‐organized bank‐like walls in the P4VP insulating layer.

Further, the spatial distributions of the ZCIS/ZnS QDs inside the inkjet‐printed spots were investigated using a fluorescence microscope (Figure 3b). As shown in the figure, the fluorescence image exhibits highly uniform distribution of QDs inside the printed spots. These uniform distributions and the distinctive height profiles for the inkjet‐printed ZCIS/ZnS QDs in the P4VP layer are clear evidence of the formation of the microinlaid QD spot structure via the selective spatial etching of P4VP and the simultaneous uniform filling of QDs, similar to the inkjet‐inlaid CBP spots in P4VP shown earlier. In contrast, when ZCIS/ZnS QD spots were inkjet printed on a phase‐inseparable PMMA layer,^[^ ^59^ ^]^ the observed height profiles of the QD spots on the PMMA layer were quite different from those of the microinlaid QD spots on P4VP. The printed QD spots on the PMMA layer showed dome‐shaped and plateau‐type height profiles (diameter: ≈90 nm, height: ≈20 nm), analogous to those of QD spots on a bare glass substrate (diameter: ≈80 nm, height: ≈15 nm, Figure S3, Supporting Information). These height profiles and the fluorescence image of the QD spots on the PMMA layer indicate that the PMMA‐based inkjet‐printing process acted only as an adding/piling tool for QDs but not as an etching/subtracting tool for the underlying PMMA layer. These results thus nicely demonstrate that the well‐defined and high‐quality patterns of the microinlaid ZCIS/ZnS QD spots in the P4VP layer were simply fabricated by the single‐inkjet‐printing process studied here.

It is noteworthy that the controlled hydrophobicity of the functional QDs realized by coating with ligands may be an important factor promoting the phase‐separation behavior between the QDs and insulating layers. Therefore, the single‐inkjet‐printing technique with using phase‐separable material combinations can be a useful and powerful tool with which to fabricate high‐quality microinlaid spot arrays for various light‐emitting devices, such as inkjet‐printed OLEDs and/or QD‐LEDs (or nano‐LEDs).

### 2.4 Device Performance of μ‐OLEDs with Inkjet‐Inlaid EML Spots

**Figure 4:**
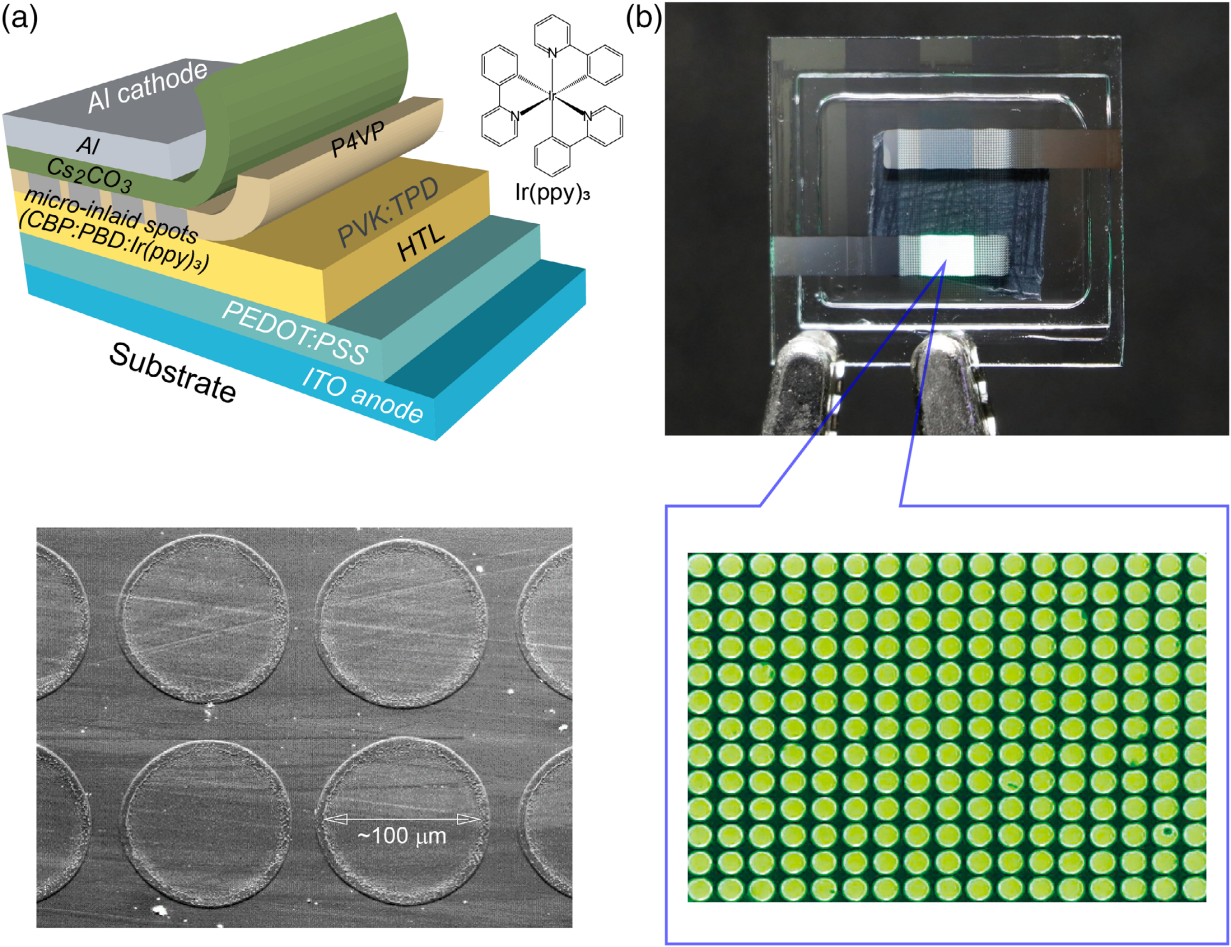
a) Schematic illustration of μ‐OLED pixels with inkjet‐inlaid EML spots of CBP:PBD:Ir(ppy)_3_ in an insulating P4VP layer (upper) and SEM image of inkjet‐inlaid EML spots in a P4VP layer (lower). b) Photograph of a green‐emitting μ‐OLED pixel array (upper, active area: 3 mm × 2 mm) and a microscopy image of the light‐emitting pixels (lower) at an applied voltage of 5.0 V. Microinlaid EML spots were fabricated with a resolution of ≈180 dpi.

Given the unique formation of the self‐organizing microinlay structure, we next turn our attention to the fabrication of μ‐OLED pixel arrays using the single‐inkjet‐printing process. For the fabrication of μ‐OLED pixel arrays, the functional ink containing CBP was reformulated by adding two more small‐molecular organic semiconducting materials: an electron‐transporting cohost of 2‐(4‐*tert*‐butylphenyl)‐5‐(4‐biphenylyl)‐1,3,4‐oxadiazole (PBD) and a green‐emitting guest of phosphorescent tris(2‐phenylpyridine)iridium(III) (Ir(ppy)_3_), together with an additional solvent of 1,2‐dichloroethane (see details in Experimental Section**)**. It should be noted that the solubility properties of PBD and Ir(ppy)_3_ are similar to those of CBP (Table S1, Supporting Information). Using this ink as a green‐emitting ink, ink droplets were single inkjet printed (≈150 pl per droplet) on a P4VP layer which was precoated on top of stacked functional layers (HTL/HIL) on an ITO substrate with HTL of *N,N′*‐bis(3‐methylphenyl)‐*N,N′*‐diphenylbenzidine (TPD)‐doped PVK (PVK:TPD) and HIL of poly(3,4‐ethylenedioxythiophene):poly(styrenesulfonate) (PEDOT:PSS) (upper panel of **Figure** **4**a). The printed sessile droplet then dissolved the underlying P4VP layer, and the convective flows of the solvents pushed the dissolved P4VP polymers from the center to the contact line of the drop, as mentioned earlier. At the same time, the evaporation of the solvents in the droplet induced a Marangoni flow, which delivered the functional solute molecules of CBP, PBD, and Ir(ppy)_3_ from the edge to the center region of the droplet. Thus, after the solvents in the droplet evaporated completely, microinlaid spots of CBP:PBD:Ir(ppy)_3_ were self‐organized on the HTL through the P4VP layer in the form of patterned EML spot pixels with an inner diameter of ≈100 μm (lower panel of Figure 4a). Next, to complete the μ‐OLED fabrication process, a Cs_2_CO_3_ electron‐injection layer (EIL) and an Al cathode were thermally deposited over the patterned microinlaid EML spot pixels. Hence, the device structure of the fabricated μ‐OLED was [ITO anode/PEDOT:PSS HIL/PVK:TPD HTL/microinlaid EML spots of CBP:PBD:Ir(ppy)_3_ through P4VP insulating layer/Cs_2_CO_3_ EIL/Al cathode] (Figure 4a). For comparison, a reference device with a conventional spin‐coated EML on a PEDOT:PSS HIL was also fabricated. Figure 4b presents a photograph of a green‐emitting μ‐OLED pixel array operating at an applied voltage of 5.0 V. The microscopy image shows that every spot pixel of the μ‐OLED is perfectly well separated and isolated from the others with a pixel spacing of ≈140 μm (≈180 dpi), even without any additional process for the typical preformation of bank‐like walls. As also clearly shown in the figure, due to the P4VP polymer laterally phase separated from the printed EML spots, the electroluminescent (EL) emission is very bright only at the on‐pixel locations and clearly dark outside of the pixels without any misalignment issues.

**Figure 5:**
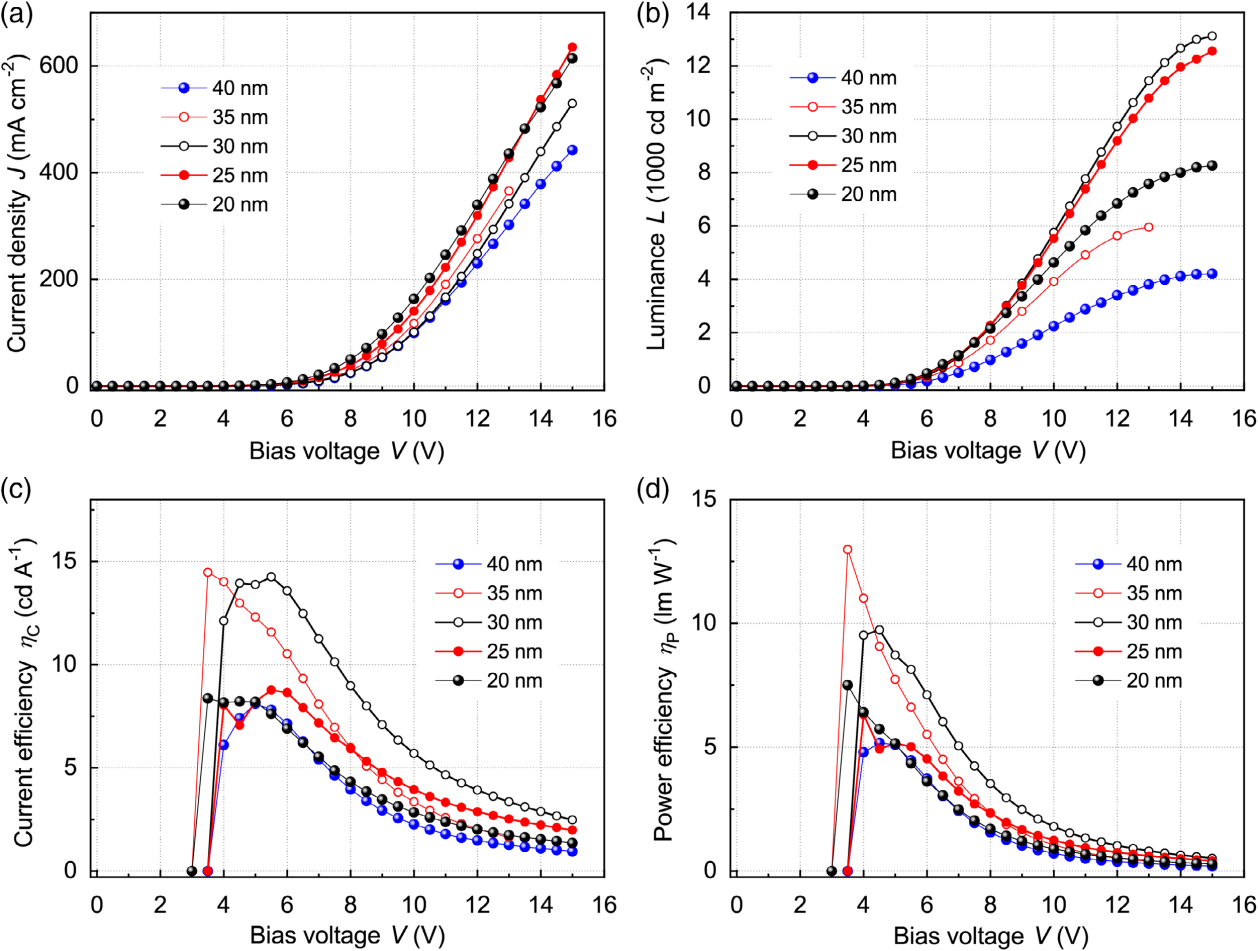
a) Current density–bias voltage (*J*–*V*), b) luminance–bias voltage (*L*–*V*), c) current efficiency–bias voltage (*LE*–*V*), and d) PE–bias voltage (*PE*–*V*) characteristics at several different thicknesses of the P4VP insulating layer.

Next, the device characteristics of the single‐inkjet‐printed μ‐OLED arrays were investigated as follows. **Figure** **5**a,b shows the observed current density–voltage (*J*–*V*) and luminance–voltage (*L*–*V*) characteristics, respectively, for μ‐OLED arrays with several different thicknesses of the insulating P4VP layer. It is clear from these figures that the *J*–*V* and *L*–*V* curves of the μ‐OLED arrays exhibit excellent diodic behaviors with steep slopes, indicating efficient charge carrier injections well below 4.0–5.5 V. For example, the operating voltage of a μ‐OLED array with a 30 nm‐thick P4VP layer is about 5.1 V for a brightness level of 100 cd m^−2^; it is 6.9 V for 1000 cd m^−2^ and 12.2 V for 10 000 cd m^−2^. The maximum luminescence reached ≈13 100 cd m^−2^ at 15.0 V. Moreover, the device efficiencies are quite high (Figure 5c,d); the best overall performance levels achieved are a peak luminance efficiency (LE) of 14.2 cd A^−1^ and a peak power efficiency (PE) of 9.7 lm W^−1^. Even at a luminance level of 1000 cd m^−2^, the LE and PE values of the array device reached 11.3 cd A^−1^ and 5.0 lm W^−1^, respectively. These device characteristics are much better than those of the spin‐coated reference OLEDs, exhibiting maximum brightness of ≈1,800 cd m^−2^ and a peak efficiency of ≈4.4 cd A^−1^ (Figure S4, Supporting Information). Moreover, the device characteristics of the single‐inkjet‐printed μ‐OLED arrays exceed those of previous inkjet‐printed devices fabricated using the intrinsically phase‐inseparable polymer PMMA,^[^ ^44^ ^]^ as expected. It is also noteworthy that the green μ‐OLED array studied here showed much higher brightness levels even with comparable peak efficiency values as compared with those of conventional well‐optimized inkjet‐printed OLED devices with additional hole‐blocking and electron‐transport layers.^[^ ^28^ ^]^ Such high device performance of the μ‐OLED array indicates the effective radiative recombinations of excitons with a good electron–hole balance due to the high quality and full coverage of the EML materials inside the inkjet‐inlaid spots.^[^ ^59^ ^]^

### 2.5 Large‐Area μ‐OLEDs with Inkjet‐Inlaid EML Spots

**Figure 6:**
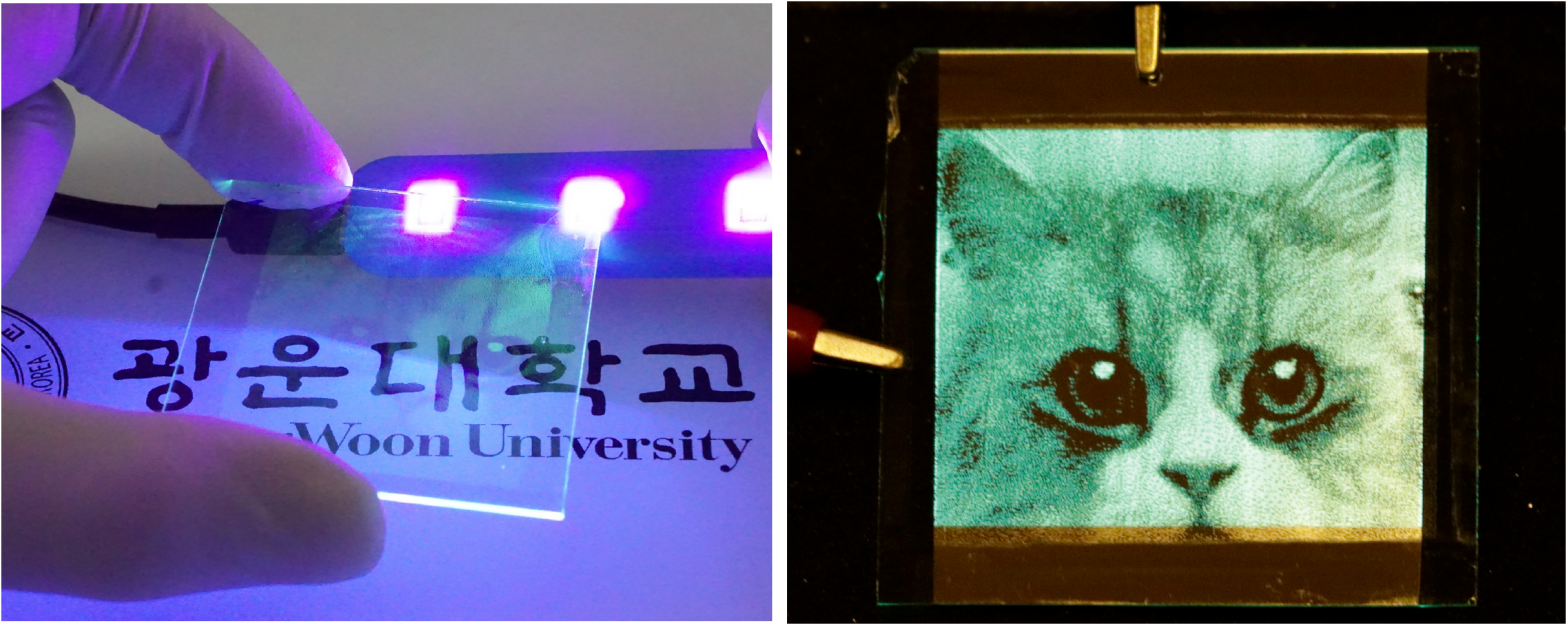
Photographs of a large‐area pattern with self‐organized microinlaid green EML spots produced by the single‐inkjet‐printing process (200 dpi), under UV light illumination (left), and a corresponding EL image of the working μ‐OLED array pattern at 6.0 V (right).

Next, encouraged by the remarkable results obtained from the μ‐OLED arrays based on the inkjet‐inlaid EML spot structure, we fabricated large‐area μ‐OLED array patterns on 6.0 cm × 5.5 cm glass substrates to assess the processability of the single‐inkjet‐printing technology. **Figure** **6** displays photographic images of operating large‐area μ‐OLED array patterns fabricated with microinlaid EML spot pixels. As shown in the figure, quite fine and sophisticated patterns of μ‐OLEDs were simply fabricated with the single‐inkjet‐printing technology. Although microinlaid EML spot pixels of μ‐OLEDs were fully single inkjet printed, the photographs clearly show that one can easily build complex patterns of highly luminous and efficient μ‐OLED spot pixels in the simplest way, in contrast to conventional complex inkjet‐printing processes. Moreover, low variation of the EL emission intensity over the entire printed area implies that there were not only very few defects such as pinholes and/or misaligned pixels but also small thickness variations in the inkjet‐inlaid EML spot pixels (see also Multimedia V1, Supporting Information). In light of these observations, it is clearly demonstrated that the single‐inkjet‐printing technique for microinlaid EML spots using the aforementioned phase‐separable material combination can be used to fabricate high‐performance light‐emitting pixel arrays with easy scaling up to larger sizes, showing considerable promise for the mass production of high‐quality and large‐area light‐emitting display devices.

Finally, we want to comment on the spatial resolution of the inkjet‐inlaid spot structures, which can be increased further more than 280 dpi using an inkjet nozzle with a small orifice diameter (e.g., ≤30 μm, Figure S5, Supporting Information). Such a high resolution of 280 dpi for these microinlaid spot patterns is already close to the pixel resolutions of currently manufacturer‐developed inkjet‐printed OLED displays.^[^ ^60^ ^]^ It is also important to note that the device performance of the inkjet‐inlaid μ‐OLEDs can be improved further by selecting optimal host/guest materials, solvents, and other parameters for different color emissions (Figure S6, Supporting Information) and/or by combining the devices with additional functional layers such as hole‐blocking or electron‐transporting layers, as reported in the literature. In addition, together with upscalable printing techniques^[^ ^61^ ^]^ such as screen printing, bar coating, blade coating, slot‐die coating, horizontal dip coating, or 3D coating for common functional layers, this simple inkjet‐printing process for light‐emitting microinlaid spot arrays can be used for the upscaling and mass production of fully printed high‐performance display devices in the near future. Furthermore, the microinlaid spot formation of functional materials realized by the single‐inkjet‐printing technique can also be put to good use in the development of new types of electronic devices, such as not only simple 2D flexible or stretchable devices but also complex conformal (3D)‐printed electronics.

## 3 Conclusion

In summary, a novel inkjet‐inlaid spot array was investigated using a material combination consisting of a phase‐separable insulating polymer and semiconducting compounds for the simple and reliable fabrication of high‐performance light‐emitting pixel arrays. It is shown that the simplest single‐inkjet‐printing process, involving simultaneous spatial etching and filling processes by microfluid flows in the sessile droplet, can implement fine pitch patterning of functional microinlay structures via the lateral phase‐separation behavior described earlier. The self‐organizing ability of microinlaid pixels inside the inkjet‐printed spots was confirmed by confocal micro‐Raman measurements, demonstrating complete demixing of the insulating polymer from the microinlaid spot structure. Moreover, it is shown that the microinlaid spot structures can be used to produce light‐emitting pixel sites with a pixel resolution exceeding 200 dpi even without any additional complex processing for the conventional preformation of bank‐like structures. For green emissions from the inkjet‐inlaid μ‐OLED arrays, relatively high maximum brightness and peak luminous efficiency levels of 13 000 cd m^−2^ and 14.2 cd A^−1^, respectively, were easily achieved. These high device performance levels are attributed from the creation of high‐quality microinlaid EML spots without any misalignment of the inkjet‐printing process, which can open up various possibilities of highly luminous, efficient, and large‐area solution‐processable small‐molecular μ‐OLEDs for diverse applications. Therefore, given its advantages of easy fabrication and fine processability, the inkjet‐inlay structures reported here will not only widen the range of the application of phase‐separable functional materials but also provide solid foundations for various semiconducting devices that have sophisticate patterns, such as inkjet‐patternable surface‐emitting devices, high‐performance solution‐processable OLED or QD‐LED (or nano‐LED) light sources or display devices, and next‐generation 2D and 3D optoelectronic devices.

## 4 Experimental Section

4.1

4.1.1

##### Materials

Poly(4‐vinylpyridine) (P4VP, number‐average molecular weight *M*_n_ = 60.0 kDa, weight‐average molecular weight *M*_w_ = 137.4 kDa), poly(9‐vinylcarbazole) (PVK), *N,N′*‐bis(3‐methylphenyl)‐*N,N′*‐diphenylbenzidine (TPD), and 2‐(4‐*tert*‐butylphenyl)‐5‐(4‐biphenylyl)‐1,3,4‐oxadiazole (PBD) were purchased from Sigma Aldrich. 4,4'‐bis(carbazol‐9‐yl)biphenyl (CBP), 2,7‐Bis(diphenylphosphoryl)‐9,9'‐spirobifluorene (SPPO13), tris(2‐phenylpyridine)iridium(III) (Ir(ppy)_3_), bis(1‐phenylisoquinoline)(acetylacetonate)iridium(III) (Ir(piq)_2_(acac)), and bis[2‐(4,6‐difluorophenyl) pyridinato‐C2,N](picolinato)iridium(III) (FIrpic) were purchased from Lumtec. Poly(methyl methacrylate) (PMMA, *M*_w_ = 950 kDa) was purchased from Microchem. Poly(3,4‐ethylenedioxythiophene):poly(4‐styrenesulphonate) (PEDOT:PSS) aqueous solution (Clevios PVP. Al 4083) used here was purchased from H.C. Starck. Hydrophobic Zn–Cu–In–S/ZnS QDs (ZCIS/ZnS, particle size: 4–5 nm, emission wavelength: 530 ± 15 nm) in powder form were purchased from PlasmaChem GmbH. All chemicals and reagents were used as received without further purification.

##### Device Fabrication

A patterned 80 nm‐thick ITO layer (30 Ω square^−1^) on a glass substrate was used as a transparent anode for the inkjet‐processed micro‐OLEDs (μ‐OLEDs). The ITO substrate was ultrasonically cleaned with ethanol, detergent, and deionized water and then dried with N_2_ gas. Just before use, the ITO substrate was treated with an ultraviolet ozone cleaner for 5 min. For the coating of HIL, the aqueous solution of PEDOT:PSS was spin coated on the ITO substrate and annealed at 120 °C for 20 min to form a 40 nm‐thick PEDOT:PSS HIL. To form an HTL, a solution of PVK and TPD (1.29 wt%, PVK:TPD = 7:6) dissolved in chloroform was spin coated on the PEDOT:PSS HIL, after which the coated film was baked at 50 °C for 2 min, yielding a 40 nm‐thick HTL on the PEDOT:PSS HIL. Then, a solution of P4VP dissolved in isopropyl alcohol (IPA, 0.5 wt% in IPA) was spin coated on the PVK:TPD HTL, yielding a 30 nm‐thick P4VP insulating layer on the HTL. Subsequently, the P4VP‐coated substrate was transferred to a nitrogen glovebox to form microspot arrays by an inkjet printer (Omnijet 100, Unijet) with a piezoelectric nozzle (MicroFeb, orifice diameter: 50 or 30 μm).

For the inkjet printing of CBP, an ink solution of CBP (1.94 wt%) dissolved in chloroform as a solvent was prepared. For the inkjet printing of the QDs, an ink suspension of ZCIS/ZnS QDs (0.34 wt%) dispersed homogeneously in a solvent mixture of chloroform and 1,2 dichloroethane (7:1) was prepared. For the green‐emissive ink for the μ‐OLEDs, an ink solution of CBP, PBD, and Ir(ppy)_3_ (1.94 wt%) dissolved in a blend of chloroform and 1,2‐dichloroethane was prepared.

Using the prepared inks, ink droplets were printed on top of the P4VP‐coated substrate by applying a driving voltage signal with a trapezoidal pulse (pulse length: 27 μs, applied voltage amplitude: *V*_pp_ = 40 V) to the nozzle of the inkjet printer at a printing velocity of 500 mm s^−1^ between the ejections of two subsequent droplets. The inkjet‐printed spot array on the substrate was baked at 70 °C for 3 min. Finally, a 2 nm‐thick Cs_2_CO_3_ EIL and a 100 nm‐thick Al cathode layer were deposited onto the top of the inkjet‐printed substrate via thermal vacuum deposition at a rate of ≈0.15 nm s^−1^ under a base pressure below 2.0 × 10^−6^ Pa. The light‐emitting active area of the fabricated μ‐OLED devices was 2 × 3 mm^2^.

##### Film and Device Characterization

To investigate the surface morphologies of the fabricated layers and spots, variations in their surface roughness levels were monitored using a noncontact 3D optical surface profiler (NV‐2400, Nanosystem Co. Ltd.). The inkjet‐printed spots and patterns were also investigated using a confocal Raman microscope (Alpha 300, WITec, spectral resolution: 1 cm^−1^, lateral spatial resolution: 200 nm). The current density–luminance–voltage (*J*–*L*–*V*) characteristics and the Commission Internationale d'Éclairage (CIE) chromatic coordinates were measured using a luminance meter (CS‐2000, Konica Minolta) and a power source meter (Keithley 2400). The EL spectra were recorded using a spectrometer (Ocean's Optics). Characterization of the fabricated device was carried out at room temperature under ambient conditions with simple encapsulation using a UV‐curable epoxy and a glass cap containing a moisture getter.

## Conflict of Interest

The authors declare no conflict of interest.

## Supporting information

Supplementary Material

## Data Availability

Research data are not shared.
