# Supplementary material for: Light‐Emitting Microinlaid Spots Produced through Lateral Phase Separation by Means of Simple Single‐Inkjet Printing
Source: Small Sci. 2022 May 9;2(7):2200017. doi: 10.1002/smsc.202200017 (PMC11936002; doi:10.1002/smsc.202200017)
Supplement: Supplementary file 1 — Supplementary Material [file SMSC-2-2200017-s001.zip › Fixed_supporting information_SS_20220328.pdf]

Supporting Information

Light-Emitting Micro-Inlaid Spots Produced through Lateral Phase-Separation by Means of Simple Single-Inkjet Printing

Byoungchoo Park[1,2], Jaewoo Park[1,2], Wonsun Kim[1], Seo Young Na[1], Yoon Ho Huh[1], Mina Kim[1], and Eun Ha Choi[1,2]*

Prof. Dr. B. Park, J. Park, W. Kim, S. Y. Na, Dr. Y. H. Huh, M. Kim, Prof. Dr. E. H. Choi
[1]Department of Electrical and Biological Physics, Kwangwoon University, Wolgye-Dong, Seoul 01897, South Korea
[2]Department of Plasma-Bio Display, Kwangwoon University, Wolgye-Dong, Seoul, 01897, South Korea
E-mail: bcpark@kw.ac.kr

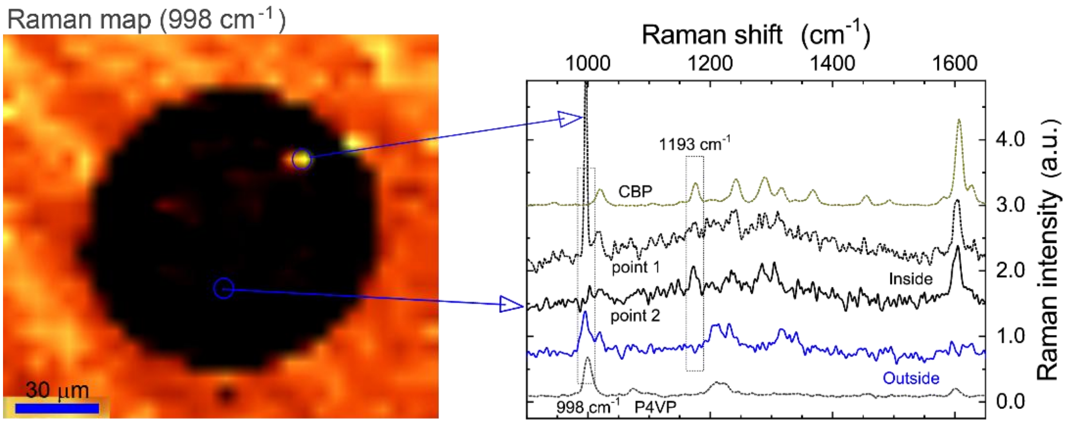

Figure S1. Left: confocal micro-Raman image of the single-inkjet-printed CBP spot in the P4VP layer, shown in Figure 2b, obtained by integrating the Raman intensities in the ring-breathing mode (998 cm^{-1}) of pyridine groups in P4VP. Right: Raman spectra at the small and bright top-right point (point 1) inside the circular dark area in the Raman image, showing a noise spike in the spectra. For comparison, the Raman spectra at the other point (point 2) inside the dark area are also shown in the figure.

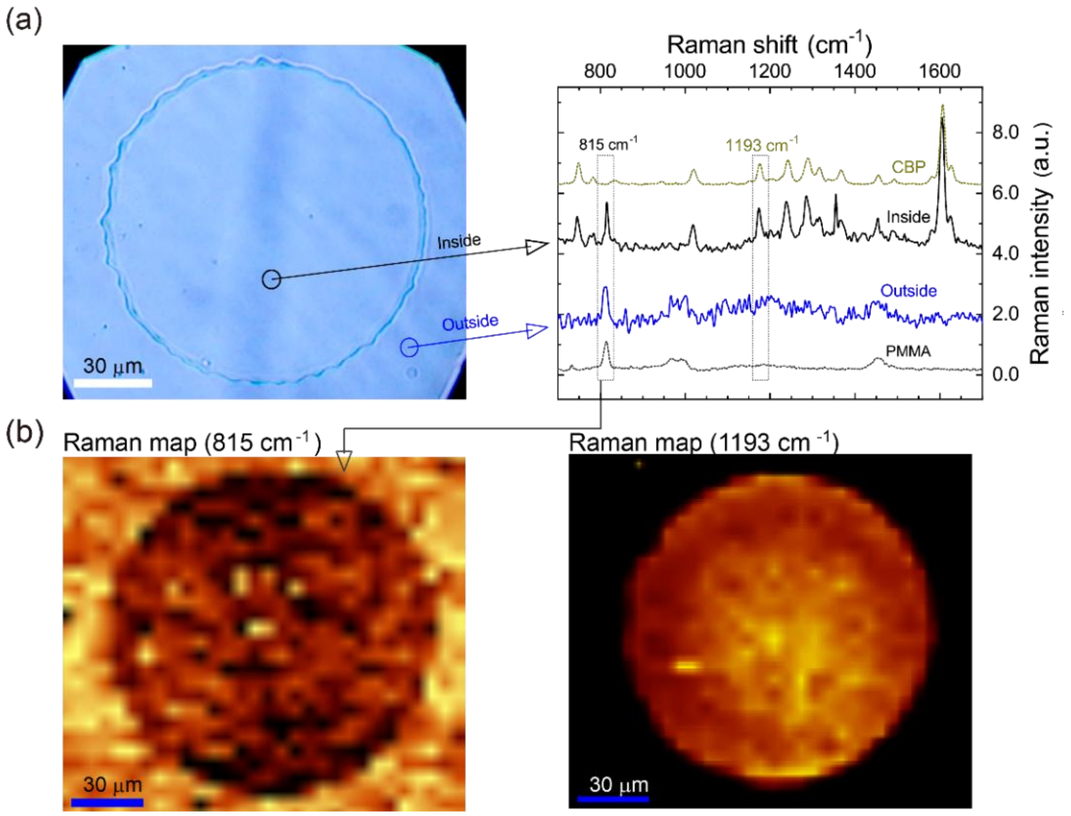

Figure S2. a) Left: optical microscope image of an inkjet-printed spot on an insulating PMMA layer using an ink containing small-molecular CBP in chloroform. Right: confocal micro-Raman spectra from inside and outside of the printed spot under 632.8 nm excitation (5 mW). For comparison, the Raman spectra from pure compound layers of CBP and PMMA are also shown in the figure. b) Raman images of the inkjet-printed spot obtained by integrating the Raman intensities for the symmetric stretching mode (815 cm^{-1}) of the C–O–C bond[1] in PMMA (left image) and for the in-plane deformation mode (1193 cm^{-1}) of the C–H bond[2] in CBP (right image).

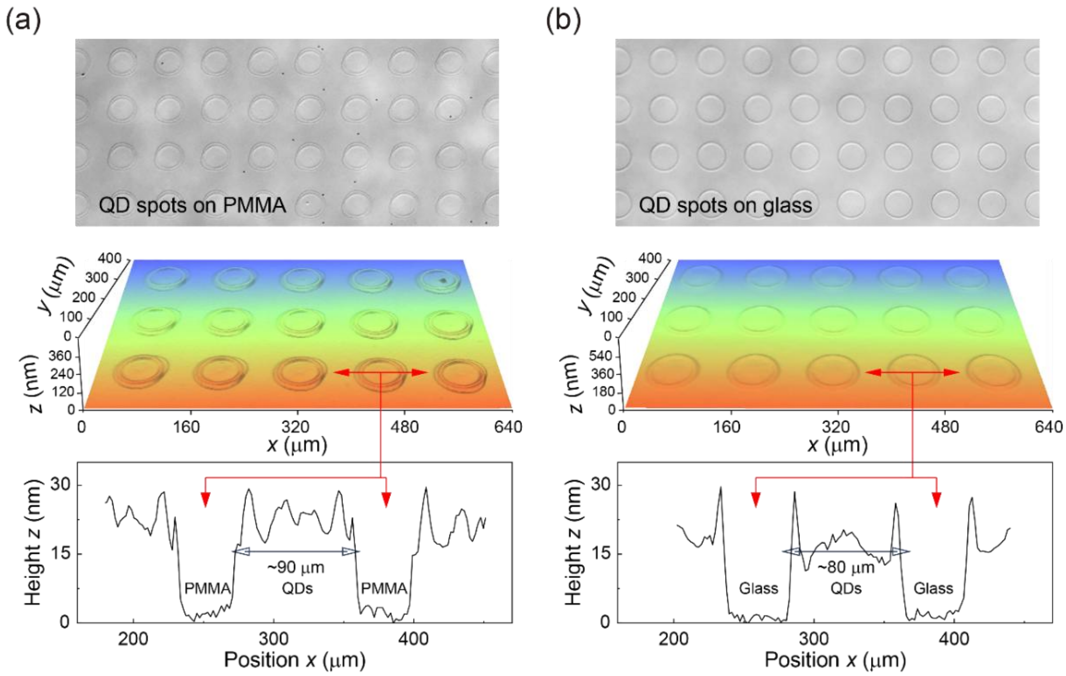

Figure S3. Optical microscope image (upper), 3D surface image (middle), and 2D profile (lower) of a rectangular spot array of Zn-Cu-In-S/ZnS (ZCIS/ZnS) core/shell QDs at 200 dpi created by the inkjet-printing process (orifice diameter: 50 μm): a) on a PMMA layer (30 nm), or b) on bare glass.

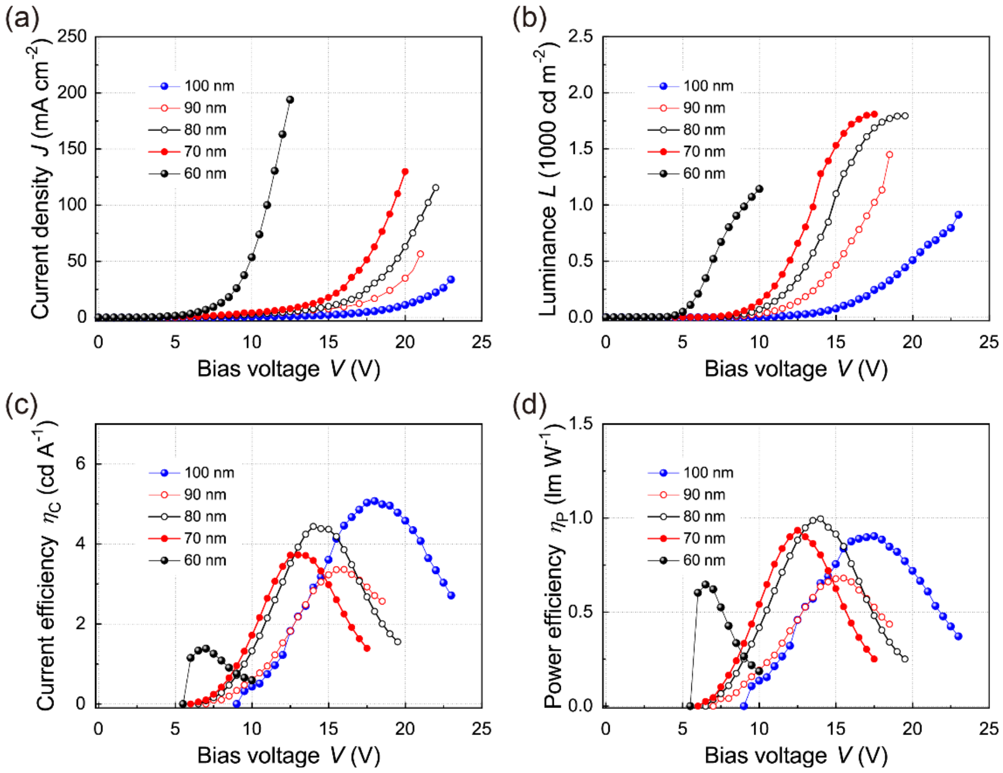

Figure S4. a) Current density–bias voltage (*J*–*V*), b) luminance–bias voltage (*L*–*V*), c) current efficiency–bias voltage (*LE*–*V*), and d) power efficiency–bias voltage (*PE*–*V*) characteristics at several different thicknesses of the spin-coated EML in reference OLEDs.

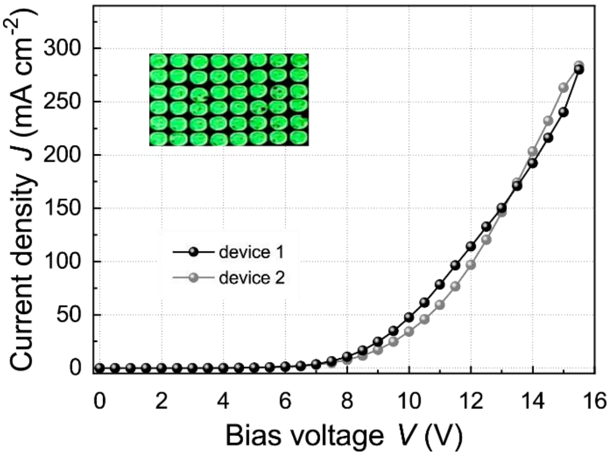

Figure S5. Current density–bias voltage (*J*–*V*) characteristics of green-emitting inkjet-inlaid μ-OLED pixel arrays with a pixel resolution of ~280 dpi fabricated using an inkjet-nozzle (orifice diameter: 30 μm). The inset in the figure shows a microscope image of μ-OLED pixels operating at 5.0 V, demonstrating high-resolution and uniform EL light emission from the well-separated circular μ-OLEDs. (Further studies of fully optimized high-resolution light-emitting devices will be reported elsewhere.)

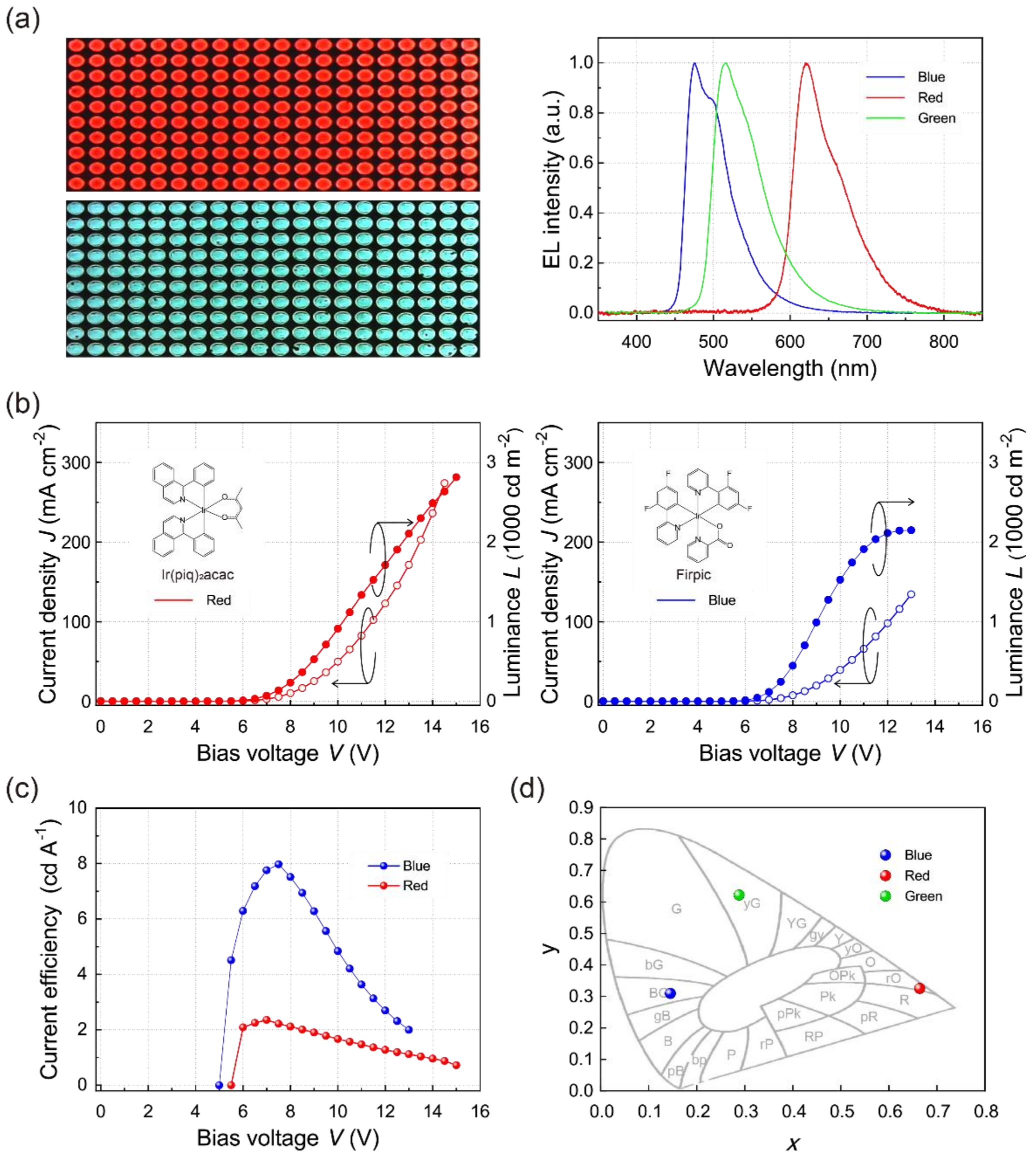

Figure S6. a) Left: Microscope images of operating red (upper) and blue (lower) μ-OLED pixels at 5.0 V, demonstrating uniform EL light emissions from well-separated circular inkjet-inlaid EML spot pixels. Right: Normalized EL spectra obtained from red, green, and blue μ-OLED pixel arrays with micro-inlaid EML spots. b) *J*–*L*–*V* and c) *LE*–*V* characteristics of red and blue μ-OLED arrays. d) The 1931 CIE chromaticity diagram of the EL emissions from the R, G, and B μ-OLED arrays studied here.

In order to obtain multi-color emissions, we also investigated two different μ-OLED pixel arrays based on inkjet-inlaid EML spots fabricated by single-inkjet printing, similar to green-emitting μ-OLEDs (Figure 4). For red emission, a guest emitter of phosphorescent bis(1-phenylisoquinoline)(acetylacetonate) iridium(III) (Ir(piq)$_2$(acac)) was introduced into the green-emissive ink at a concentration of 0.15 wt%, while for blue emission, 2,7-Bis(diphenylphosphoryl)-9,9'-spirobifluorene (SPPO13) and phosphorescent bis[2-(4,6-difluorophenyl)pyridinato-C2,N](picolinato) iridium(III) (FIrpic) were used as a host and a

guest emitter, respectively, at a concentration of 0.07 wt% in blue ink. Left panel of Figure S6a shows microscope images of the fabricated red- and blue-emitting μ-OLED arrays operating at 5.0 V. As clearly indicated in the images, well-separated circular pixels in the μ-OLED arrays emitted bright and uniform EL light. The EL spectra obtained from the red, green, and blue μ-OLED arrays showed single dominant peaks at 621 nm for the red devices, at 515 nm for the green devices, and at 476 nm for the blue devices (right panel of Figure S6a, Supporting Information), corresponding to the emissions from $Ir(piq)_2(acac)$, $Ir(ppy)_3$, and FIrpic, respectively.[3,4]

Next, we investigated the *J–L–V* characteristics of the red and blue μ-OLED arrays. As shown in Figure S6b, in both μ-OLED arrays, the charge carrier injections were well below the voltage of 4.5–5.5 V, with sharp increases in the *J–L–V* curves above this voltage range. For example, the operating voltages of the red μ-OLED array were approximately 7.3 V for a brightness of 100 cd m^{-2}, 10.3 V for 1000 cd m^{-2}, and 12.7 V for 2000 cd m^{-2}, with the luminescence reaching ca. 2800 cd m^{-2} at 15.0 V (left panel). For the blue μ-OLED array, the operating voltage was approximately 6.9 V for a brightness of 100 cd m^{-2}, while these values were 9.0 V for 1000 cd m^{-2} and 11.4 V for 2000 cd m^{-2}, with the luminescence in this case reaching ca. 2150 cd m^{-2} at 13.0 V (right panel). Moreover, the efficiency levels of the devices are relatively high (Figure S6c, Supporting Information); in the red μ-OLED array, the best overall performance was obtained with a peak *LE* of 2.4 cd A^{-1} and a peak *PE* of 1.1 lm W^{-1}. In the blue μ-OLED array, the best overall performance was obtained with a peak *LE* of 8.0 cd A^{-1} and a peak *PE* of 3.9 lm W^{-1}. Notably, these device performance levels are also fairly high and/or comparable to those of conventional well-optimized devices with typical inkjet-printed EMLs.[3] These are mainly attributed to the high-quality light-emitting EML spots, possessing quite homogeneous and smooth active areas with few defects and relatively low surface roughness levels (ca. 0.43 nm) in the inkjet-printed spots without any misalignment issue.

Note also that even at a high luminance level of 1000 cd m^{-2} for the R, G, and B μ-OLED arrays, there were small variations in their CIE coordinates of (0.66, 0.33), (0.29, 0.62), and (0.15, 0.31), respectively, showing good color stabilities of the EL emissions from the micro-inlaid EML spot pixels (Figure S6d, Supporting Information). These results show that the inkjet-inlaid EML spots produced by the simple single-inkjet-printing process are quite suitable for obtaining bright and efficient R, G, and B color emissions. It is also important to note that the device performance of the inkjet-inlaid μ-OLEDs can be improved further by selecting optimal host/guest materials and/or by combining the devices with additional

functional layers such as hole-blocking or electron transporting layers. (Further studies of fully optimized multi-color light-emitting devices will be reported elsewhere.)

Table S1. Comparison of the solubility parameters (δ) determined by the Solver Add-in of Microsoft Excel[5] for the functional compounds used in this study.

Compound	δ_D ($MPa^{0.5}$)	δ_P ($MPa^{0.5}$)	δ_H ($MPa^{0.5}$)	δ ($MPa^{0.5}$)
PBD	17.8	8.3	8.2	21.2
$Ir(ppy)_3$	18.0	12.3	7.2	23.0
$Ir(piq)_2(acac)$	18.0	12.3	7.2	23.0
SPPO13	17.2	9.9	10.5	22.4
FIrpic	18.5	10.6	9.2	23.2

δ_D: dispersion solubility parameter; δ_P: polar solubility parameter; δ_H: hydrogen bonding solubility parameter. $\delta = (\delta_D^2 + \delta_P^2 + \delta_H^2)^{0.5}$

References

[1] C. S. Reddy, A. Zak, E. Zussman, *J. Mater. Chem.* **2011**, *21*, 16086.

[2] G. Zerbi, S. Sandroni, *Spectrochim. Acta* **1968**, *24A*, 483.

[3] L. Liu, D. Chen, J. Xie, J. Piao, Y. Liu, W. Wang, K. Cao, S. Chen, *Org. Electron.* **2021**, *96*, 106247.

[4] L. Duan, L. Hou, T.-W. Lee, J. Qiao, D. Zhang, G. Dong, L. Wang, Y. Qiu, *J. Mater. Chem.* **2010**, *20*, 6392.

[5] M. Díaz de los Ríos, E. H. Ramos, *SN Appl. Sci.* **2020**, *2*, 676.
